# Integrated Quantitative Evaluation of Spatial Cognition and Motor Function with HoloLens Mixed Reality

**DOI:** 10.3390/s24020528

**Published:** 2024-01-15

**Authors:** Kenya Tada, Yuhei Sorimachi, Kyo Kutsuzawa, Dai Owaki, Mitsuhiro Hayashibe

**Affiliations:** Neuro-Robotics Lab, Department of Robotics, Graduate School of Engineering and also with Graduate School of Biomedical Engineering, Tohoku University, Sendai 980-8579, Japan; tada.kenya.p3@alumni.tohoku.ac.jp (K.T.); kutsuzawa@tohoku.ac.jp (K.K.); owaki@tohoku.ac.jp (D.O.)

**Keywords:** mixed reality, HoloLens2, game-based rehabilitation, cognitive ability, motor function

## Abstract

The steady increase in the aging population worldwide is expected to cause a shortage of doctors and therapists for older people. This demographic shift requires more efficient and automated systems for rehabilitation and physical ability evaluations. Rehabilitation using mixed reality (MR) technology has attracted much attention in recent years. MR displays virtual objects on a head-mounted see-through display that overlies the user’s field of vision and allows users to manipulate them as if they exist in reality. However, tasks in previous studies applying MR to rehabilitation have been limited to tasks in which the virtual objects are static and do not interact dynamically with the surrounding environment. Therefore, in this study, we developed an application to evaluate cognitive and motor functions with the aim of realizing a rehabilitation system that is dynamic and has interaction with the surrounding environment using MR technology. The developed application enabled effective evaluation of the user’s spatial cognitive ability, task skillfulness, motor function, and decision-making ability. The results indicate the usefulness and feasibility of MR technology to quantify motor function and spatial cognition both for static and dynamic tasks in rehabilitation.

## 1. Introduction

The proportion of the elderly in the overall population is rapidly increasing worldwide. On a global scale, the current trajectory indicates that the population aged 65 years and above will increase from 9.3% in 2020 to approximately 16.0% by 2050 [1]. Consequently, the increasing number of people requiring treatment and rehabilitation is expected to cause a shortage of doctors and therapists in the medical and welfare fields. Currently, occupational and physical therapists attend to a single patient at a time, resulting in limited time available for the evaluation and treatment of each patient. Moreover, the assessment of a patient’s physical functionality frequently relies on the subjective judgment of the therapist. This subjective approach poses problems in terms of the accurate sharing of patient information among multiple therapists. In addition, the COVID-19 pandemic has increased the demand for telemedicine and home-based rehabilitation [2,3]. The establishment of home-based rehabilitation methods that do not require hospital visits will reduce the burden on patients and hospitals by reducing the stress due to hospital visits and decreasing the risk of infection.

Cognitive function assessment is an area of rehabilitation that requires automation and digitization. Cognitive function is essential for performing daily activities smoothly. For example, driving requires cognitive functions [4,5,6,7]. In areas with aging populations, the increase in accidents caused by the operational errors of elderly drivers has become a social problem. To reduce the number of automobile accidents among the elderly, it is important for them to correctly recognize their own cognitive functions, to be aware of whether they can drive and what precautions they should take while driving. The trail-making test (TMT), which is a paper-and-pen, desk-based method, has been used to assess cognitive function [8,9]. However, driving a car requires the ability to perceive the ever-changing surroundings in a three-dimensional world and make appropriate judgments. It is difficult to measure these abilities necessary for driving using the current desk-based TMT. In addition, the therapist must explain the task and measure the time required to complete it, making it challenging for patients to perform the task on their own. Therefore, it is necessary to develop an automated and digitalized system for evaluating cognitive function.

In recent years, many studies have been conducted on the use of gamification to maintain treatment motivation and the use of virtual reality (VR) in rehabilitation [10,11,12,13,14,15]. Gamification is the integration of game design principles within non game contexts [16]. Illustrative instances of studies within the domain of game-based rehabilitation include a balancing game for assessing the body stability using center-of-gravity information [17]. VR replaces the surrounding environment with a virtual space created by a computer, allowing users to experience the virtual world as if it were the real world. Furthermore, VR technology exhibits the advantages of result quantification via digitization and the potential for rehabilitation interventions in diverse locales. However, because the VR technology is immersive, it essentially obscures the user’s entire field of vision. Therefore, the safety of the patient’s surroundings must be ensured. In addition, some users experience a phenomenon called VR sickness involving motion-sickness-like symptoms such as dizziness, headache, and nausea [18].

From this perspective, mixed reality (MR), which combines the real and virtual worlds, has attracted considerable attention in the field of rehabilitation. MR was first proposed by Paul Milgram in 1994; everything between the real and virtual environments refers to MR, and this concept is called the reality–virtual continuum [19,20]. While conventional two-dimensional assessments such as desk-based evaluations are limited in scope, MR technology makes it possible to evaluate a wider range of three-dimensional capabilities. MR creates real-time interactions between the virtual and real-world elements, as represented by interactions such as collisions and obstacles with real-world surfaces such as floors and walls. Furthermore, MR-based interventions can be implemented in familiar settings such as hospital rehabilitation spaces or home living spaces. Because of these characteristics, ongoing research is exploring the potential of MR for the rehabilitation of both cognitive and motor functions [21,22,23,24,25]. For instance, Franca et al. [22] devised a rehabilitation task incorporating virtual objects to help people struggling with cognitive impairments. Tada et al. [25] developed a reaching task using MR to measure and evaluate motor and cognitive functions.

Nevertheless, the majority of the rehabilitation systems developed in these previous studies are digitalized versions of existing assessments and often lack a spatial component. Furthermore, most virtual objects in these systems remain static and have little interaction with the surrounding environment. However, the real world comprises countless three-dimensional entities with spatial orientations and dynamic motion. Considering the movements in daily life, rehabilitation and ability testing using dynamic objects in an environment similar to reality are considered effective. Through the digitalization of rehabilitation, MR has the potential to solve this challenge and become a method for measuring and evaluating dynamic tasks.

This study aimed to develop an evaluation and training system that uses mixed reality head-mounted display (MR-HMD) and dynamic interaction with the surrounding environment to achieve automatic measurements and quantitative evaluations of physical functions, including spatial cognition and motor skills, during rehabilitation. By developing this system, we attempted to demonstrate the effectiveness of MR-based tasks involving interaction with the environment and dynamic elements. Specifically, the following three MR applications were developed:

1. The MR-TMT application, which added spatial and dynamic components to the existing TMT neuropsychological test to assess attentional and executive functions.
2. MR-Trace application, a three-dimensional path-tracing task designed to assess spatial grasping capability, dexterity, and motor coordination.
3. MR-BallContact application, a task in which the subject responds appropriately to an approaching ball to assess reflexes and judgment.

Tada et al. [25] developed an MR task using the Tower of Hanoi and Jenga to assess cognitive function. In the study, the virtual objects used in the task are static and have little interaction with real-world objects. In contrast, this study used dynamic virtual objects for the task, which also incorporate interaction between the virtual and real-world objects. Through the development of these systems, we demonstrated the possibility of using MR as an alternative to existing tests and attempted to evaluate the patient’s abilities in a spatial environment that is closer to daily life. Furthermore, we verified the potential of MR to measure abilities that cannot be measured by existing testing methods through tasks that take advantage of real-time interaction with the real environment and dynamic tasks.

## 2. Materials and Methods

This section provides a description of the MR device used, the application developed, and an overview of the experiments performed. In the description of the applications, a detailed description of the three tasks developed is given.

### 2.1. MR Device

In this study, Microsoft HoloLens 2 [26] was employed as the MR-HMD. This device draws virtual objects through a see-through holographic lens to make objects appear as if they exist in real space, enabling a seamless overlay between the real world view and the virtual environment. HoloLens 2 is entirely wireless and autonomous and requires no connection to external devices. The integration of a 9-axis IMU sensor, depth camera, and infrared sensor enables hand and eye tracking, covering both manual dexterity and fine finger movements.

The application was developed using Unity Technology’s Mixed Reality Toolkit (MRTK), which is a cross-platform development toolkit for Unity. Foundational scripts, input systems, and user interfaces are readily available within the MRTK framework for creating MR-HMD applications.

### 2.2. Application

In this study, MR applications were created with the primary objectives of automated measurement and quantitative assessment of physical functionalities, including spatial cognition and motor function, to support rehabilitation. The devised system is self-sufficient because it relies solely on the capabilities of HoloLens 2, obviating the need for external measurement devices. The task execution and measurements were exclusively performed and recorded using the HoloLens 2 interface. For tasks that required time measurement, the time spent on the task was measured within the application. Subsequently, the gathered data were transmitted to a computer for analysis and evaluation of the examination and training outcomes. The display of the holograms and the acquisition of data through the tracking function were performed at a frame rate of 60 fps. This section provides a brief overview of the three applications developed in the study.

#### 2.2.1. MR-TMT

**Figure 1:**
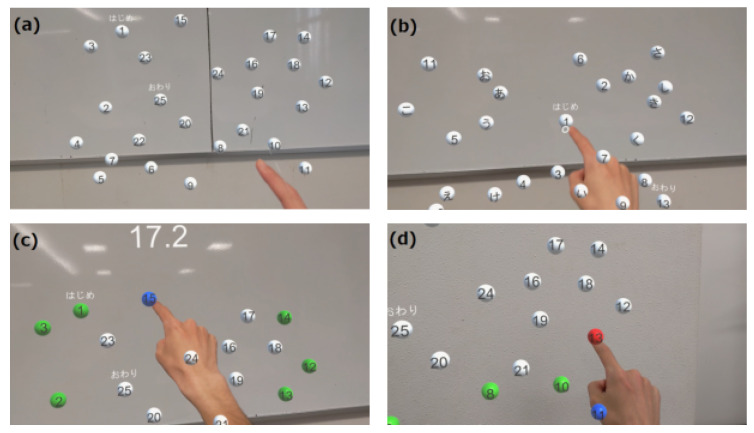
MR-TMT play screen from subject’s viewpoint. The target was a spherical white object. The elapsed time was displayed at the top of the screen. (**a**) In Part A, the object is a sphere with numbers 1–25 written on it, which are touched sequentially. (**b**) In Part B, 13 numbers from 1 to 13 and 12 hiragana from あ to し are displayed. The subject is required to touch the numbers and hiragana alternately and in order (1, あ, 2, い, 3, ..., し, 13). (**c**) Screen when touching the correct object. When the correct object is touched, its color changes from white to blue. When the next correct object is touched, the color changes from blue to green. In other words, the correctly touched objects are green and the last correctly touched object is blue. (**d**) Screen when touching the wrong object. If the wrong object is touched, it changes to red to make the subject aware of the incorrect selection.

The MR-TMT, shown in Figure 1, is a reaching task that emulates the TMT, a well-established neuropsychological assessment method. The scene of executing the MR task of this paper can be also confirmed at the video in the supplementary material. The TMT evaluates the attentional and executive dysfunctions commonly associated with dementia and higher-order cognitive impairments [8,9]. It involves connecting the targets on a paper with lines. In recent years, the TMT has been digitalized, and TMT systems [27,28,29] have been developed using tablets and VR technologies. Pinkes et al. [30] developed a real-world version of the TMT, called Can-TMT, which involves taking cans labeled with numbers or letters out of a shelf in sequence. In the MR-TMT system developed in this study, the index finger of the dominant hand was used to touch the displayed spherical object. Similar to the TMT, the MR-TMT comprises two task variants: Part A, where participants sequentially touch numbered targets from 1 to 25, and Part B, which entails alternating between numbers and hiragana (Japanese syllabary characters, e.g., 1, あ, 2, い, 3, ..., し, 13). The numbers and hiragana characters on these targets follow the conventional TMT method. The placement of its objects is point symmetrical of the conventional TMT placement. The size of the object is based on the size of the conventional TMT, but is made larger for ease of tasking. In Parts A and B, we introduced a task in which the target object moved, which was not possible in the conventional TMT. The touched objects changed color from white to green upon successful interaction, where incorrect touch caused the objects to turn red. The task completion time, frequency of touch errors, and number of instances when the fingertips extended beyond the object plane constituted the evaluative metrics for MR-TMT.

1. Task completion time: The duration taken to successfully touch all 25 objects from task initiation.
2. Number of missed touches: The number of erroneous touches on objects other than the intended targets.
3. Reaching count: The count of instances where the fingertips exceeded the plane where the objects were situated.

Part A primarily requires visual perceptual skills, whereas Part B primarily requires working memory and the ability to switch between tasks [31]. In this application, we verified whether the static task could produce the same evaluation results as the conventional TMT test, and whether the dynamic task could evaluate abilities different from those of the conventional TMT. In the experiment, the MR-TMT and TMT were conducted and results were compared. We also recorded and discussed the gaze information that could not be obtained with the conventional method and the touch errors that occurred when the task was performed in space instead of on a desk.

#### 2.2.2. MR-Trace

**Figure 2:**
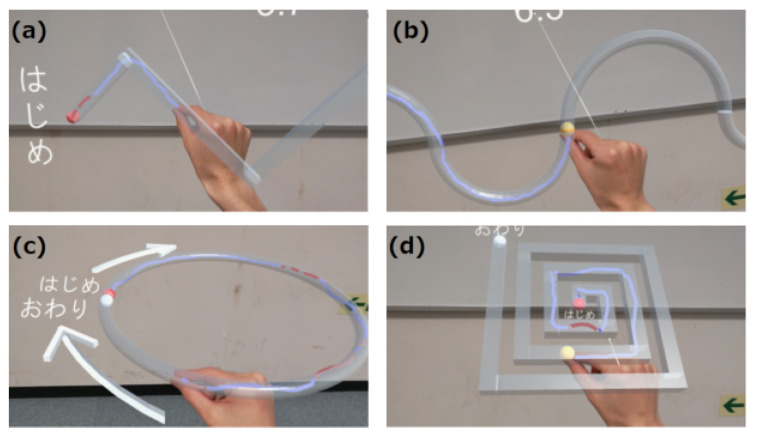
Screen of MR-Trace task. The translucent gray patterns in the image show the target paths to be traced. The target paths are (**a**) zigzag, (**b**) wave, (**c**) circle, and (**d**) spiral. The starting point is the red spherical object and the ending point is the white spherical object. The subject starts at the red sphere and traces through the target path to the white sphere using the fingertip. The fingertip trajectory is displayed in blue if the trajectory is within the path and in red if it goes outside the path. (**a**,**c**) comprise the Trace task, whereas (**b**,**d**) are the Track task in which a yellow spherical object moves at a certain speed, and the user is required to move the fingertip in accordance with the movement of the yellow spherical object.

MR-Trace is a drawing task that uses 3D objects to assess motor functions such as spatial grasp, dexterity, and motor coordination (Figure 2). In this context, “dexterity” refers to the ability to perform mentally conceived complex tasks and movements with precision. “Coordinated movement”, on the other hand, refers to the synchronization of multiple body components, leading to fluid and harmonious movements. The task is divided into two parts: “Trace”, where the subject traces the presented object at their own pace, and “Track”, where the subject traces the object at a pre-determined pace.

In the tracking task, a spherical object moves uniformly across the traced trajectory from the start to the endpoint. A virtual line can be drawn using the thumb and index finger, with the tip of the index finger serving as the drawing point. The trajectory appears as a three-dimensional structure and accurate tracking is required to ensure that the index finger fits within the frame. The fingertip trajectory is drawn in blue if it is within the path and in red if it is outside the path, providing visual feedback to the user. There are four different path types for this task: “zigzag” and “wave” paths lie in the frontal plane, “circle” paths reside in the horizontal plane, and “spiral” paths have a 45° inclination relative to the horizontal plane. For straight paths, the cross-section has a square shape, with each side measuring 2 cm, whereas a circular cross-section with a diameter of 2 cm is employed for curved paths. The four path shapes were set up to evaluate performance at different curvatures.

MR-Trace utilizes the following six quantitative evaluation indices:

1. Task Completion Rate: This is an indicator of the percentage of the fingertips on the object and the accuracy of the tracing process, and is measured using the following metrics.
  (1) $$\frac{T_{\mathrm{in}\;\mathrm{object}}}{T_{\mathrm{completion}}}$$
  $T_{\mathrm{in}\;\mathrm{object}}$ indicates the number of frames in which the fingertip is on the path, and $T_{\mathrm{completion}}$ indicates the total number of frames until the end of the task.
2. Path efficiency: Expresses the ratio of the shortest path length to the actual distance traversed by the fingertip. This indicator is expressed using
  (2) $$\frac{D_{\mathrm{actual}}}{D_{\mathrm{shortest}}};$$
  $D_{\mathrm{actual}}$, which is the actual path distance traveled by the fingertip; and $D_{\mathrm{shortest}}$, which is the shortest distance in the path;
3. Average external distance: The mean distance from the object wall when the fingertip lies outside the object, serving as an indicator of the magnitude of errors. This can be expressed as follows:
  (3) $$\frac{\sum_{t=1}^{T}d_{A}\left(a_{t}\right)}{T_{\mathrm{out}\;\mathrm{object}}}.$$
  *t* is the number of frames from the start of the task, $a_{t}$ is the fingertip coordinate at frame *t*, A is the path, $d_{A}\left(a_{t}\right)$ is the distance between the fingertip $a_{t}$ and path A, and $T_{\mathrm{out}\;\mathrm{object}}$ is the number of frames in which the fingertip is outside the path;
4. Average Deviation: This index indicates the degree of deviation from the center of the path throughout the task and is expressed as follows:
  (4) $$\frac{\sum_{t=1}^{T}d_{l}\left(a_{t}\right)}{T_{\mathrm{completion}}}.$$
  *l* is the centerline of the path and $d_{l}\left(a_{t}\right)$ is the distance between the fingertip $a_{t}$ and the centerline of the path *l*;
5. Maximum Deviation: Indicates the maximum deviation from the center of the path in each task.
  (5) $$\max d_{l}\left(a_{t}\right);$$
6. Distance from a moving point (evaluation index for Track only): The average distance between fingertip of the index finger and the location of a moving spherical object. It indicates the extent of deviation from the designated pace and is expressed as follows:
  (6) $$\frac{\sum_{t=1}^{T}d_{b_{t}}\left(a_{t}\right)}{T_{\mathrm{completion}}}.$$
  $b_{t}$ denotes the coordinates of the *t* frame moving point at frame *t*, and $d_{b_{t}}\left(a_{t}\right)$ is the distance between the moving point $b_{t}$ and fingertip $a_{t}$.

MR-Trace is a drawing task in space and, unlike drawing on a flat surface, it requires spatial cognition skills because the depth of objects must be taken into account. The pathways are arranged in frontal planes, horizontal planes, and diagonal orientations, each of which requires different arm movements. Detailed quantitative evaluation may be possible because the trajectory of the fingertips is recorded.

#### 2.2.3. MR-BallContact

**Figure 3:**
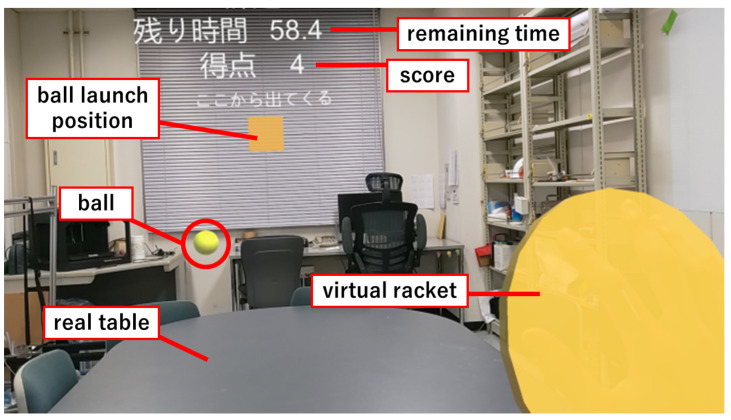
MR-BallContact play screen from subject’s viewpoint. When the right hand is recognized, a yellow disk-shaped virtual racket is displayed over the hand. A yellow cube object is displayed at the center of the screen. From this object, a ball of each color is launched in a random direction. The balls bounce on the real desk and fly toward the player. Scores are gained by touching the flying ball with the virtual racket. The remaining time of the game and the score (number of balls touched) are displayed at the top of the screen.

The MR-BallContact task is to react to a flying ball and hit the ball to a virtual racket. This task uses a real desk. In this task, a virtual ball is launched toward the player from a designated point in the field of view (Figure 3). Utilizing the spatial recognition capabilities of HoloLens 2, the ball bounces off the surface of the physical desk and comes within arm’s reach of the player. The player then hits the ball with a virtual racket that appears in his or her hand to score points. When a player interacts with the ball, visual and auditory feedback is provided through changes in color and sound.

This application performs two types of tasks. The first task involves launching two balls with varying repulsion coefficients. The balls exhibit different colors, with the yellow ball possessing a higher repulsion coefficient than the blue ball. These balls are projected horizontally with a consistent initial velocity in random directions, exclusively on the left and right sides. The task duration spans 90 s, during which the balls are launched at random intervals of 2–4 s. Diminishing the intervals and reducing the preparation time makes the task more arduous, indicating the capacity of the subject to make immediate decisions during the test. The evaluation indicators used in this task are as follows.

1. Percentage of balls not touched.
2. Percentage of balls touched that should not have been touched (Only the second task).

This task requires the subject to recognize the color of the ball and the direction in which it is moving, to predict its trajectory, and to decide whether to touch or dodge the ball. Therefore, reflexes and the ability to make instantaneous judgments are required. In this study, the number of misses and eye movements were also recorded and examined.

### 2.3. Experimental Details

The three developed applications were tested on ten healthy young adults without cognitive or motor impairments. Given that each application was executed independently, the participants varied based on the application. The participants were provided with instructions on operating the MR-HMD and were given sufficient time to familiarize themselves with the MR environment. The experimental procedures were conducted with the subjects in a seated position. Following the completion of the measurements, the subjects were interviewed to gauge their perspectives on the applications and their experiences with the MR-HMD.

In addition to the developed MR-TMT, we conducted the traditional TMT as a means of comparison with the MR-HMD test. The TMT was conducted before the MR-TMT.

## 3. Results

**Figure 4:**
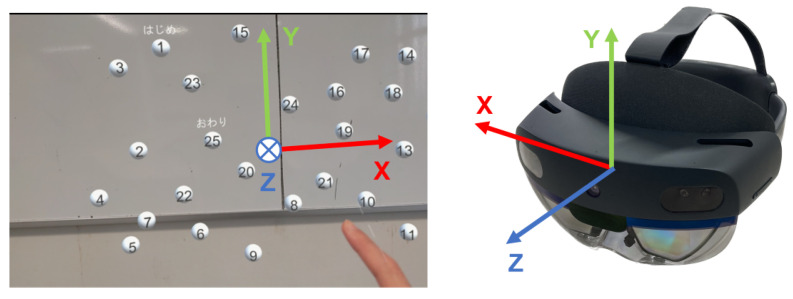
Coordinate system of HoloLens 2 application developed with unity. The origin of the coordinates is the position of HoloLens 2 when the application is launched. The x-, y-, and z-axes point to the right, upward, and frontal directions, respectively, as seen by the HoloLens 2 wearer.

This section describes the results of the experiments conducted for each application. The left-hand coordinate system used in the graphs is the same as that used in Unity. The origin of the coordinates is the position of HoloLens 2 when the application is launched. The x-, y-, and z-axes point to the right, upward, and frontal directions, respectively, as seen by the HoloLens 2 wearer (Figure 4).

### 3.1. Comparison between MR-TMT and TMT

Ten male participants, with an average age of 23.5±1.3 years and consisting of 8 right-handed and 2 left-handed individuals successfully completed all tasks. The mean completion times for the TMT and MR-TMT are presented in Table 1. To assess the statistical significance, Wilcoxon’s signed rank test was conducted on the completion times of Part A and Part B for TMT, MR-TMT (static), and MR-TMT (dynamic), as well as on the static and dynamic tasks (Part A and Part B of MR-TMT). The significance level was set at 5%. The TMT completion times were found to be comparable to the age-specific durations documented by Tombaugh [9] (Part A averaged 22.93±6.87 s and Part B averaged 48.97±12.69 s for the 18–24 years age group). This suggests that the participants’ attentional and executive functions were within the normal range. In the MR-TMT, completion times for both the static and dynamic tasks were longer for Part B, which is consistent with the trend observed in the TMT. From the Wilcoxon’s signed rank test, there was a significant difference in the completion time between Parts A and B for both the static and dynamic tasks. However, the B/A ratios (ratio of the completion time of Part B to that of Part A) for MR-TMT were 1.35 (static) and 1.49 (dynamic) in contrast to 2.08 for TMT, indicating a distinct increase in time. In comparing the static and dynamic tasks, the completion time for the dynamic task exceeded that of the static task for both Part A and Part B. However, this difference did not reach statistical significance.

**Figure 5:**
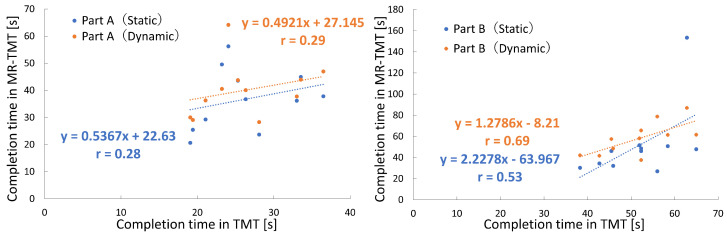
Relationship of MR-TMT completion time to TMT.

Figure 5 shows the completion times of the TMT and MR-TMT for each subject. A weak positive correlation was observed between the completion times of TMT Part A and MR-TMT Part A, with correlation coefficients of 0.28 for the static task and 0.29 for the dynamic task. In Part B, the correlation coefficient between the TMT and MR-TMT static tasks was 0.53, whereas that between the TMT and MR-TMT dynamic tasks was 0.69, indicating positive correlations. These results indicated that subjects with longer TMT completion times tended to have longer MR-TMT completion times.

Table 2 shows the average number of errors for 10 subjects in both the TMT and MR-TMT tasks. In TMT, the error is represented by the number of times a subject connected an incorrect letter, whereas in MR-TMT, the error is measured by the number of missed touches. The average number of missed touches was higher in the dynamic task than in the static task, and the results of the Wilcoxon signed rank test showed a significant difference between MR-TMT Part A (static) and MR-TMT Part A (dynamic).

Table 3 presents the average number of reaching attempts made by the subjects in MR-TMT. The number of times reached includes the number of times the object was touched correctly (25 times), the number of times it was missed, and the number of times the subject unsuccessfully attempted to touch the object. An increase in the number of attempts to reach the object was observed between the static and dynamic tasks, and a statistically significant difference was detected in Part B according to the Wilcoxon signed rank test. In the static task, the average number of missed touches was 0.3 in Part A and 0.4 in Part B. The average number of times the fingertip passed without touching any object was 6.1 in Part A and 4.3 in Part B. This value was obtained by subtracting the number of times the correct object was touched (25 times) and the number of missed touches from the number of reaching actions. Similarly, in the dynamic task, the average number of missed touches was 2.0 in Part A and 1.4 in Part B. The average number of times the fingertip passed without touching any object was 6.5 in Part A and 9.2 in Part B.

**Figure 6:**
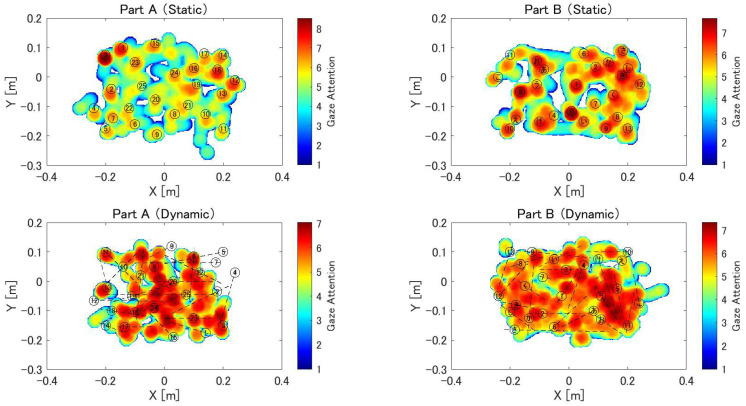
Heat map of eye gaze during MR-TMT. The heat map consists of a 300 × 400 mesh of 2 mm per side. In each frame, values from 0 to 12 are added to the mesh within a circle with a radius of 25 mm from the gazing point, depending on the distance from the gazing point. This is repeated until the end of the task, and the natural logarithm of the value of each mesh is displayed as a heat map. Locations with longer gaze times are shown in red, and those with shorter gaze times are shown in blue. The numbers and hiragana in the figure correspond to the positions where the object was displayed during the task.

Figure 6 shows the heat maps that quantitatively represent the subject’s gaze during task performance. These are the data obtained from one of the ten subjects. The numbers and hiraganas in the figure correspond to the object locations in the task. In the heat map of the dynamic task, the initial object position is indicated, and the black dashed line indicates the trajectory of the object as it moves. Overall, the static task causes a red area around the object, whereas the heat map of the dynamic task causes the entire area to appear red.

### 3.2. MR-Trace

A total of 10 male subjects (eight right-handed and two left-handed) with a mean age of 23.5±1.3 years performed the drawing task on MR-Trace. The drawing movements were recorded. Subsequently, we calculated several evaluation indices for each movement, including the task completion rate, path efficiency, average external distance, average deviation, maximum deviation, and distance from a moving point.

**Figure 7:**
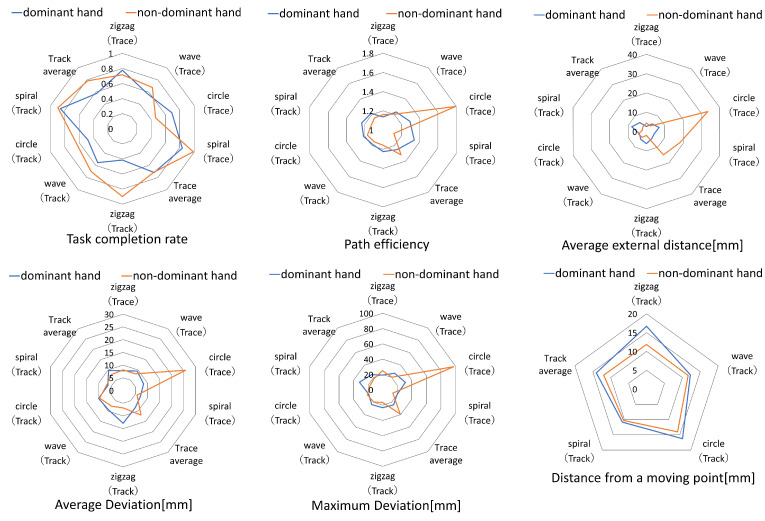
Score for each object shape.

Figure 7 displays the radar charts of one subject, presenting the results obtained for each evaluation index for each task. These charts display the outcomes for four object shapes (zigzag, wave, circle, and spiral) in both the Trace and Track tasks, along with the average values for each task. The color blue denotes the scores pertaining to the dominant hand, whereas orange represents the non-dominant hand. A higher value for the task completion rate indicates better performance. For the remaining five indicators, lower values indicate a better performance.

### 3.3. MR-BallContact

MR-BallContact was performed on 10 subjects (age 23.0±1.3 years, 10 males, 9 right-handed, and 1 left-handed).

Table 4 presents the error rates observed in each subject’s performance. The yellow results of Task 1 and Task 2 correspond to omission errors, whereas the pink results of Task 2 represent commission errors. In task 1, 10.5% of the subjects made omission errors. Furthermore, on average, the subjects exhibited a higher error rate when interacting with the blue ball. Task 2 resulted in 5.0% omission errors and 2.0% commission errors for the subjects as a whole.

**Figure 8:**
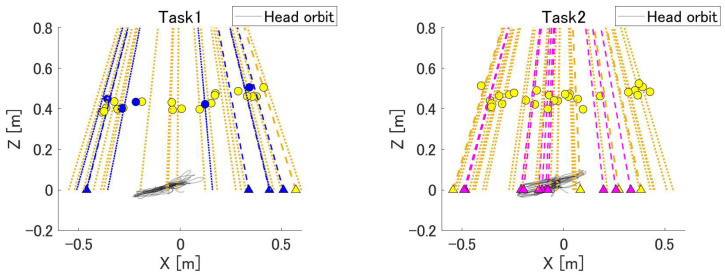
Ball ejection direction and head trajectory. The circles in the figure represent the coordinates of the palm of the hand when the ball is touched, and their color corresponds to the color of the ball. Each dotted line represents the predicted trajectory of the ball. Triangles represent balls that could not be touched; these are drawn at the intersection of the ejection direction and z = 0 m. The gray lines indicate the head coordinates during the task.

Figure 8 shows the projected directions of the ball and head trajectories generated during the experiment. The circles in the figure represent the coordinates of the palm when the ball is touched, with the color of each symbol corresponding to the color of the ball. The triangles denote balls that could not be touched; these are drawn at the intersection of the ejection direction and z = 0 m. Each dashed line represents the expected trajectory of the ball. The graph of Task 1 shows that the subject made many mistakes while the ball was flying to the right.

**Figure 9:**
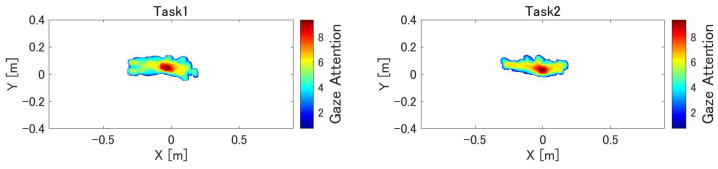
Heat map of gaze during MR-BallContact. The heat map consists of a 300 × 700 mesh of 3 mm per side. In each frame, values from 0 to 7 are added to the mesh within a circle with a radius of 20 mm from the gazing point, depending on the distance from the gazing point. This is repeated until the end of the task, and the natural logarithm of the value of each mesh is displayed as a heat map. The origin represents the point of origin of HoloLens 2, which is the position of the cube from which the ball is launched. Because the ball touching position was in the range of 0.2–0.4 m, the heat map is shown in the XY plane at z = 0.4 m.

Figure 9 presents the heat maps of the gaze direction during MR-BallContact. The heat maps indicate that the subjects gazed at the center and moved their eyes left and right in response to the flying ball.

## 4. Discussion

### 4.1. User Feedback

In this section, we discuss the effectiveness of the applications. After the experiment, many subjects answered that they enjoyed the application. Because the subjects wore the MR-HMD for only 10–15 min, none of them complained of virtual reality sickness, such as motion sickness. Negative feedback regarding the application was that the display area of the virtual environment was unexpectedly small when wearing the MR-HMD. In particular, MR-TMT and MR-BallContact require frequent head and eye movements, which may cause certain objects to be outside the display area or out of sight. In addition, the experimental results for all three applications were likely to be satisfactory because the experiments were conducted on young people with no impairment of cognitive and motor functions. Further experiments with older participants are required to verify these scores.

Because hand tracking was performed using a camera mounted on HoloLens 2, it is not possible to acquire the joint positions when the hand is outside the recognition range of the camera. In this experiment, the coordinates of the fingertips could not be recorded in some cases because the arms were extensively stretched up, down, left, and right. This is an unavoidable problem because of the specifications of HoloLens 2 and the display range of the virtual image. It is important to develop MR applications that fully utilize the currently available functions and performance levels while looking forward to future advances in MR technology.

### 4.2. MR-TMT

Task completion times for MR-TMT were longer for Part B than for Part A by 14.9 s for the static task and only 18.1 s for the dynamic task. This pattern matches that in the conventional TMT assessment. In Part B, the correlation coefficients between TMT and MR-TMT task completion times were 0.53 for the static task and 0.69 for the dynamic task, both positive correlations. This implies that the MR-HMD assessment is proficient in quantifying cognitive modalities, namely attentional and executive functions, similar to the traditional paper-and-pen test. The ratio of task completion time for Part B to Part A (herein referred to as B/A) was 2.08 for the standard TMT and 1.35 for the MR-TMT. This was partly due to the size of the inspection area. The conventional TMT is confined to the dimensions of an A4 sheet of paper, measuring 29.7 cm in height and 21 cm in width. Conversely, the MR-TMT projects an object onto an area twice the size of that used in the conventional TMT (33 cm long and 50 cm wide). Hence, B/A was smaller owing to the increased distance and time for arm movement.

The difference was not significant, but the completion time for dynamic tasks was 3.3 s in Part A and 6.5 s in Part B, longer than the completion time for static tasks. As shown in Table 2, the number of missed touches for the dynamic task was 1.7 for Part A and 1.0 for Part B, higher than for the static task. It should be noted that, in Part A, there was a marked variation in the missed touch. Furthermore, the number of reaching for the dynamic task was 2.1 for Part A and 5.9 for Part B, higher than for the static task. The increase in error frequency in Part B was due to the increased complexity of the task as a result of the incorporation of transitional elements between numerals and kana characters. Owing to the perpetual motion inherent in the dynamic task, even if subjects recognized the object before the order of touch, they would have moved to a different location when they actually touched it. Furthermore, touching requires the prediction of the trajectory of the moving object and finger. Therefore, dynamic tasks demand capabilities of motion prediction and spatial discernment, which in turn amplify the difficulty of the task. The three evaluation indicators used in this study may serve as indicators of the task complexity.

The heat map in Figure 6 shows the distribution of the participants’ gaze during the exercise. In the static task, the participants’ gaze was focused on the object, whereas in the dynamic task, the participants’ gaze extended over the entire observation area. In the static task, the participants spent more time looking closely around an object when touching it. By contrast, the dynamic task produced divergent responses. The subjects’ gazes showed mobility in synchrony with the trajectory of the dynamic object, resulting in red regions extending across the entire heat map. Fernandez et al. [32] reported a marked difference in eye movements during reading between patients with Alzheimer’s disease and healthy subjects. Oyama et al. [33] developed a task to diagnose dementia using eye-tracking data. Therefore, there is a possibility that eye-tracking data can be used to evaluate cognitive functions in this task as well.

### 4.3. MR-Trace

Figure 7 presents a radar chart for comparing the results of each evaluation indicator obtained for each task. Circular and spiral paths tended to score lower than zigzags and waves. This was likely due to the difference in the direction of the hand movement. The first two involved movements in the depth direction and required bending and stretching of the arms, which could easily cause misalignment with the target. In the latter two paths, the elbow joint was moved from left to right with a fixed depth and vertical movement, resulting in less elbow extension and flexion movement and therefore less misalignment.

Within the group, certain participants exhibited a superior performance in the Track task, which required pursuing a moving point, when compared with the Trace task, which demanded a circular sketching motion. This was because the presence of a moving point made it easier to grasp the distance to the object. Although the objects in MR-Trace are translucent white, it may have been difficult for subjects unfamiliar with MR to grasp their exact location. In contrast, the orange color of the moving point helped to determine its spatial location.

### 4.4. MR-BallContact

Table 4 shows the error rates for each subject. In Task 1, the blue ball (lower coefficient of repulsion) had a higher error rate than the yellow ball by 6.9%. After bouncing on the table, the yellow ball reached its highest point at about eye level, whereas the blue ball reached its highest point about 20 cm below eye level. The blue ball had a smaller bounce, making its trajectory more difficult to predict, whereas the yellow ball had a mountainous bounce, making its trajectory more predictable. In Task 2, which involved judging the balls to be touched, only three subjects made mistakes. The ball that was mistakenly touched was on a trajectory toward the subject’s own body; hence, it is possible that the subject touched the ball without being able to avoid it.

Figure 8 shows the direction of ball ejection and the position of the hand at the time of touch and describes the bias in the trajectory of the ball that could not be touched by each subject. A quantitative understanding of the strong and weak trajectories of each subject can lead to more efficient training. If the error rate for the ball is high at any angle, it can be concluded that the player is unable to predict the trajectory of the ball and perform an appropriate reaching motion. If there is a bias in the location of the ball that could not be hit, the player may not be able to move the hand in that direction properly or may not be able to see the ball because of a bias in the field of vision. This can be determined by examining the heat map of the line of sight. Thus, it is possible to infer the factors that affect the subject’s ability and score from the tendency to make mistakes and gaze at the information.

However, because only one ball was ejected at a time in this task, the reaching decision in Task 2 could be easily made by simply looking at the color of the ball. The experiment was conducted on young subjects, and the results showed that commission errors did not occur for many of them. If multiple balls are projected simultaneously from multiple locations, the task becomes more complex and requires instantaneous judgment and action. Such a challenging setup would have resulted in greater differences in the results between the subjects. The heat map of eye movement shows that the subjects moved their eyes left and right more than up and down because the ball was flying at an angle to the left and right.

### 4.5. Limitation

A limitation of this study was that only ten subjects were tested for each application, which did not generate a sufficient amount of data. The subjects were in their early 20s and had high cognitive and motor functions. Therefore, future studies should focus on collecting data from elderly subjects with declining abilities and subjects with disabilities in cognitive and motor functions to demonstrate the practicality of the proposed system. It is necessary to explore the possibility of differences in the measurement results according to age and problems related to task difficulty or user interface. Although we were able to demonstrate that MR-TMT can measure cognitive and physical functions similar to existing tests, we were not able to sufficiently validate the measurement results of the other two applications. It is necessary to verify whether the applications can reliably measure the subjects’ abilities to assess their usefulness for actual rehabilitation. The applications can be used for evaluation as well as training. Therefore, it is also necessary to confirm the effectiveness of MR-HMD over a long period to determine whether the patients’ scores increase or their symptoms improve after training for a certain period of time.

## 5. Conclusions

In this study, three MR-HMD-based applications were developed to automatically and quantitatively evaluate human spatial cognition and motor function. This approach includes three-dimensional dynamic tasks and tasks that interact with the surrounding environment, which have rarely been performed in conventional testing or previous studies. Each application was tested on young subjects; the usefulness of the applications in the MR environment was examined and the measurement results were discussed. It was suggested that the MR-TMT, which mimics the TMT, an existing attentional and executive function assessment test, measures cognitive functions (attentional and executive functions) similar to the conventional method. MR-Trace, a tracing task of a three-dimensional virtual object, quantitatively expressed spatial comprehension, spatial movement skill, fine motor coordination, and left–right differences based on the differences in object shapes. MR-BallContact is a task in which participants touch a virtual ball that flies randomly and interacts with a real desk. In this application, which requires instantaneous prediction of the trajectory of the ball as it moves from moment to moment and reaching to the appropriate position, the tendency of error for each subject was obtained. The MR-HMD system can record various quantitative data such as fingertip, head, and gaze trajectories, enabling us to assess the cognitive and physical abilities of the subjects. A disadvantage of MR-HMD is that the viewing angle of the displayed virtual environment is smaller than that of the actual field of vision. However, several subjects who participated in the experiment had favorable opinions regarding the task using the MR-HMD. The use of MR, which can maintain a realistic field of vision, makes it easier to perform rehabilitation tasks in the home environment when compared with training or testing using VR technology. Another advantage is that MR-HMD tasks can be performed alone without the help of a therapist. By installing multiple applications on the MR-HMD, the need to prepare equipment for each examination or training session can be reduced, which also allows more time for treatment. It is also easy to change the level of difficulty and measure the dynamic tasks according to the subject’s level of difficulty. Thus, rehabilitation using MR-HMD reduces the burden on both patients and therapists, and has the potential to quantitatively measure abilities that could not be measured using conventional methods. It is expected that various rehabilitation methods using MR technology will be realized in the future.

## Figures and Tables

**Table 1:** Comparison of TMT and MR-TMT completion times (subject average).

	TMT	MR-TMT (Static)	MR-TMT (Dynamic)
Part A [s]	26.3 ± 6.2	36.8 ± 11.9	40.1 ± 10.7
Part B [s]	51.9 ± 8.8	51.7 ± 36.8	58.2 ± 16.2
B/A	2.08 ± 0.61	1.35 ± 0.50	1.49 ± 0.42

**Table 2:** Number of touch misses (subject average).

	TMT	MR-TMT (Static)	MR-TMT (Dynamic)
Part A	0.3 ± 0.5	0.3 ± 0.7	2.0 ± 1.5
Part B	0	0.4 ± 0.8	1.4 ± 1.4

**Table 3:** Number of reaching (subject average).

	MR-TMT (Static)	MR-TMT (Dynamic)
Part A	31.4 ± 3.9	33.5 ± 4.6
Part B	29.7 ± 2.7	35.6 ± 6.1

**Table 4:** Error rate for each subject.

	Task 1			Task 2	
	**Total [%]**	**Yellow [%]**	**Blue [%]**	**Yellow [%]**	**Pink [%]**
Subject 1	17.2	5.0	44.4	12.5	0
Subject 2	13.8	15.8	10.0	0	0
Subject 3	6.7	5.9	7.7	0	6.7
Subject 4	13.3	11.1	16.7	0	0
Subject 5	6.7	14.3	0	0	0
Subject 6	10.0	16.7	5.6	6.3	8.3
Subject 7	13.3	0	28.6	3.0	0
Subject 8	3.6	0	5.0	3.1	0
Subject 9	3.3	0	4.8	3.8	0
Subject 10	17.2	8.3	23.5	21.7	5.0
Average	10.5 ±5.0	7.7 ±6.2	14.6 ±13.1	5.0 ±6.7	2.0 ±3.1

## Data Availability

Data are contained within the article.

## Supplementary Materials

The following supporting information can be downloaded at: https://www.mdpi.com/article/10.3390/s24020528/s1.
